# Epilepsy in patients with Angelman syndrome

**DOI:** 10.1186/1824-7288-36-31

**Published:** 2010-04-16

**Authors:** Agata Fiumara, Annarita Pittalà, Mariadonatella Cocuzza, Giovanni Sorge

**Affiliations:** 1 Pediatric Neurology, Regional Referral Centre for Inborn Errors Metabolism, University of Catania, Italy; 2 Child Neuropsychiatry, Department of Pediatrics, University of Catania, Italy

## Abstract

Angelman syndrome (AS) is a neuro-behavioural, genetically determined condition, characterized by ataxic jerky movements, happy sociable disposition and unprovoked bouts of laughter in association with seizures, learning disabilities and language impairment. Most of the cases are hardly diagnosed during infancy as jerky movements, the cardinal sign, appear later in childhood.

AS is caused by a variety of genetic mechanisms involving the 15q 11-13 chromosome. About 70% of cases are due to a "de novo" interstitial deletion in the long arm region, arising on the maternally inherited chromosome. The diagnosis is confirmed by methylation test or by mutation analysis of UBE3A gene. The deletion phenotype is generally linked to a more severe clinical picture in that 95% of patients manifest more severe seizures, severe mental and motor retardation, dysmorphic features and microcephaly.

The pathogenesis of epilepsy in AS is still not fully understood. The presence in the commonly deleted region of a cluster of genes coding for 3 subunits of the GABAa receptor complex has lead to the hypothesis that GABA neurotransmission is involved.

Epilepsy, often severe and hard to control, is present in 85% of patients within the first three years of life, although less than 25% develop seizures during the first year. It was observed that febrile seizures often precede the diagnosis. Most frequent types are atypical absences, generalized tonic-clonic, atonic or myoclonic seizures, with multiple seizure types occurring in 50% of deleted patients. There is still some doubt about the association with West syndrome.

The EEG abnormalities are not themselves pathognomonic of AS and both background activity and epileptic discharges vary even in the same patient with time. Nevertheless, the existence of some suggestive patterns should facilitate the early diagnosis allowing the correct genetic counselling for the family. Some drugs seems to act better than others, Valproate, ethosuximide and clonazepam giving the best results.

## Introduction

Angelman syndrome (AS) is a neuro-behavioural, genetically determined condition, described in 1965 by Dr. Harry Angelman, a British paediatrician[1].

Patients are now widely known for their behavioural and motor pattern well defined as "happy puppet", although this denomination is actually avoided because of the possible derision meaning to the family. These children indeed present ataxic jerky movements, happy sociable disposition and unprovoked bouts of laughter, in association with seizures, learning disabilities and language impairment[2]. Characteristic facial features include deep set eyes, pointed chin, macrostomia, wide-spaced teeth, and brachycephaly. Blond hair and blue eyes can appear unusual when compared to the familial phenotype.

The estimated frequency is 1/15000 - 1/20000 [3]. In 1995, Williams et al.[4] established the clinical criteria for the diagnosis.

**Figure 1:**
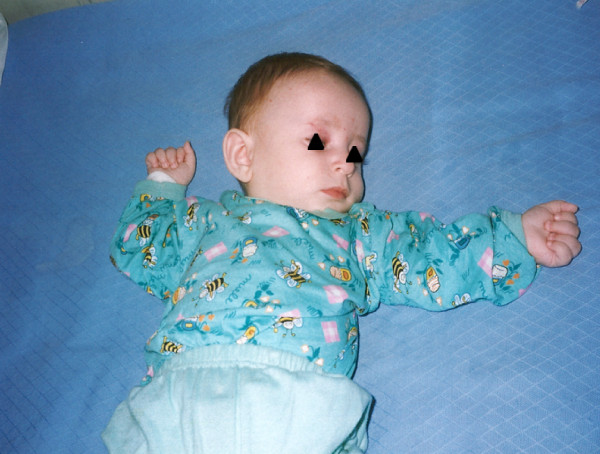
**Subtle finger tremors**. A five months old boy with Angelman syndrome and drug resistant infantile spasms.

Most of the cases are hardly diagnosed during infancy when the facial appearance can be not yet clear and subtle signs as hands and fingers tremor, easy smile and feeding difficulties are generally underestimated (Fig. 1). With time, in early childhood, delayed milestones, without appropriate language development, deceleration of head growth, epilepsy and sleep disorders become evident. The appearance of the typical jerky movements, the cardinal sign of late childhood picture, facilitates the diagnosis.

## Genetics

AS is caused by a variety of genetic mechanisms involving the 15q11-13 chromosome. About 70% of cases are due to a "de novo" interstitial deletion, involving the long arm region of the maternally inherited chromosome[5]. In a low percentage (3%) AS is linked to a paternal uniparental disomy (UPD) in that the child has inherited both chromosomes 15 from the father[6]. AS may also derive from imprinting defects (ID) during gamethogenesis [7]. Another genetic mechanism was identified in the occurrence of mutations of the maternal UBE3A gene[8]. This gene encodes the E6AP-3A ubiquitin protein ligase involved in brain protein degradation[9]. Maternally imprinted in the brain, it is expressed in cerebellum Purkinjie cells, olphactory tracts and hippocampus[10]. UBE3A anomalies are present in 85% of AS with familial recurrence and 15 -23% of sporadic ones. De novo mutations occur in 70% of cases. A high incidence of gonadal mosaicism has also been observed interesting both maternal and paternal germline.

The clinical diagnosis is confirmed by methylation test or, when this is negative, by the mutation analysis of UBE3A gene. Nevertheless, in a small percentage (5-10%) of subjects with a clinical diagnosis of AS, any cytogenetic or molecular alteration is found[11]. These patients could bear a not yet identified genetic anomaly involving UBE3A activity or even have a wrong clinical diagnosis.

The deletion phenotype is generally linked to a more severe clinical picture in that 95% of patients manifest a worse seizures pattern, severe mental and motor retardation, dysmorphic features and microcephaly[12]. Patients with UPD are less severely affected so that the diagnosis is often suspected later[11]. Generally they reach walking abilities, some interaction with the environment is observed and only 20% of them present seizures. Patients with imprinting anomalies have a even milder course with less seizures and better communication skills[13]. UBE3A mutation patients are similar to deletion patients as far as concerns seizures, microcephaly and absence of speech.

## Pathogenetic Hypoteses

The pathogenesis of epilepsy in AS is still not fully understood. The presence in the commonly deleted region, 15q11-q13, of a cluster of genes coding for 3 subunits of the GABAa receptor complex has lead to the hypothesis that GABA neurotransmission is involved in AS[14].

All the involved genes are greatly expressed in several areas of the embryonic and newborn brain. Anyhow, the animal model, obtained by blocking these genes, shows signs different from the clinical AS pattern and conversely almost 30% of AS patients are not deleted in that specific region.

UBE3A gene codes for a ubiquitin protein ligase acting in a pathway where proteins are marked to reach specific proteasomes. Ubiquitin related enzymes are known to increase during growth-factor induced neuronal differentiation. Thus ubiquitin-mediated proteolysis might be involved in posttranslational processing of some precursor proteins that play a role in synaptogenesis and at synaptic receptors level[9]. GABAa receptor is connected with a chloride channel and acts inhibiting neurotransmission. All these aspects can have a deep impact on a developing brain and the forthcoming epilepsy although the exact pathogenic effect is still unknown.

Patients with 15q11-q13 microdeletion generally show more severe neurologic pictures. In the animal model a thalamocortical dysfunction resulting from dysregulation of synaptic GABAergic transmission was observed. This finding might explain the typical EEG rhythmic pattern seen in patients with AS [14].

The phenotypic differences between patients with 15q11-q13 microdeletions and those with imprinting defect or UPD suggest that in the latter group the residual expression of the gene can allow a less severe clinical picture [15].

Recent studies have stressed the overlapping manifestation of other genetic syndromes such as RTT. Samaco et al.[16] suggested a possible role of MeCP2 in the regulation of UBE3A and GABRB3 expression in mammalian brain thus increasing the awareness of a possible link between these two conditions.

## Seizures

Seizures are present in 85% of patients within the first three years of life[17], although less than 25% develop seizures during the first year[18]. Onset of seizures has been described at various ages, as early as one month up to 20 years[19].

Epilepsy is often severe. All types of seizures have been described and in most cases they are hard to control and recur in clusters, alternating with seizures free periods. Most frequent types are atypical absences, generalized tonic-clonic, atonic or myoclonic seizures, with multiple seizure types occurring in 50% of deleted patients. Among the main ictal pattern, are also complex partial seizure, mainly occipital, [20] together with clonic unilateral seizures[21].

It was observed that febrile seizures often precede the diagnosis of AS[22] and that even moderate temperature increases show a triggering effect[23]. Based on their own experience, Galvan-Manso et al[24] refer that myoclonic seizures are the most frequent type at onset (as observed in 25% of their patients between 4 months and 5 years), followed by atonic seizures (23%). Generalized tonic clonic seizures (21%) and atypical absences (12%) were observed around 3 years of age. Less frequently, seizures at onset, in their series, were extension spasms (9%) within the 1st year of age, flexion spasms (5%) at 6 months and focal seizures (5%) at 1 year.

Pelc et al[25] stress that, despite the widespread opinion, epileptic spasms are uncommon. Infantile spasms are typical of West syndrome and are characteristically associated with so called "hypsarrhythmic" EEG. Although flexion or extension spasms are reported in AS, the EEG pattern is not frankly hypsarrhythmic consisting of runs of 2-3 c/s of high amplitude higher than 300 μV. A number of reports described the occurrence of Lennox-Gastaut syndrome but the runs of slow spike-waves complexes seen in AS are usually rhythmic with a non-convulsive status epilepticus.

Periods of reduced contact, reduction of motor activity and cognitive impairment, suggestive of neurological regression, might instead hide episodes of non-convulsive or not detectable status epilepticus. Indeed, Valente et al[22] registered status epilepticus (SE) in 15/18 (84%) patients, myoclonic SE in 7/18 (15%). Guerrini et al[21] consider the peculiar limbs tremor the epiphenomenon of cortical myoclonus. Recently Elia [26] by polygraphic studies, documented that myoclonic jerks are not correlated with EEG paroxysmal abnormalities. This "*myoclonic status in non progressive encefalopathies (MSNE*)" is characterised by the association of absences, subcontinuous jerks and brief myoclonic absences. This pattern can be observed also in other genetic conditions such as Prader-Willi, Wolf-Hirschhorn and Rett syndrome (RTT). Moreover, like in Rett syndrome, in AS seizures are often difficult to detect and distinguish from other hyperkinetic movements. Indeed, sometimes, the differential diagnosis between girls with AS and those with RTT can be difficult. Stereotyped hand movements, microcephaly, absence of speech and poorly controlled seizures, are shared signs. Periodic breath spells, hyperventilation, small and cold hands and feet are more suggestive for a diagnosis of RTT, but in some cases the final word is reserved to the cytogenetic and molecular investigations[27].

## EEG

The EEG does not show a standard pattern of anomalies and both background activity and epileptic discharge vary even in the same patient.

Nevertheless, some suggestive EEG anomalies and epileptic crises can allow a early diagnosis.

Since 1988, Boyd et al[28] described three typical patterns commonly observed in AS patients and present both in the awake and in the sleeping state, regardless of the clinical evidence of seizures:

1. persistent generalized rhythmic 4-6 Hz activity not influenced by eye closure (differing from what is seen in other conditions, where such stimulations succeed in blocking the activity; it should be helpful in the differential diagnosis for patients younger than 12 years).

2. rhythm delta activity of 2-3 Hz more evident in the anterior regions, while spikes and sharp waves represent the inter-ictal discharge pattern. Moderate amplitude and multifocal spikes appear during epileptiform activity. Generalised slow activity is predominant.

3. spikes and sharp waves mixed with 3-4 Hz components of amplitude higher than 200 μV mainly from the posterior area and triggered by eye closure.

These patterns appear early in life, from the 4^th ^month of life, so they represent a precious tool for the early diagnosis. With time EEG abnormalities change and, according to their experience and previous reports[11,19,23], Laan et al[29] describe high voltage slow burst activity of 1-3 Hz mixed with high amplitude 4-6 Hz activity in children younger than 4 years, passing to 4-6 Hz activity from posterior regions mixed with spike and waves in following years till puberty. Adult patients show a very slow background rhythm, at times mixed with focal or multifocal spikes.

The same Authors[29], in their series of 36 AS patients, found 50% of them showing an intermittent and sometimes continuous run of rhythmic triphasic 2-3 Hz activity of high amplitude (200 - 500 μV) mainly from the frontal regions and mixed with spikes or sharp waves areas. Characteristically these triphasic waves occurred in alert patients while in other conditions (i.e. metabolic diseases) they are generally observed in comatous patients.

In 45% of his patients with 15q11-q13 deletion and 30% of those with UBE3A mutation, Elia [26] described MSNE characterised by a slow background activity, subcontinuous theta-delta activity involving the central areas, or brief sequences of rhythmic delta waves with superimposed spikes mainly involving the parieto-occipital area and activated by eye-closure.

There is still some concern about the association of AS and West syndrome.

Matsumoto et al[19] described young AS patients with hypsharrhythmic EEG based on the finding of irregular delta waves associated with moderate to high amplitude epileptiform discharges with multifocal distribution. Neurophysiological studies on larger series of patients, made by Dan and Boyd[14], demonstrated that the EEG pattern commonly seen in AS can be easily differentiated from hypsharrhythmia, consisting of runs of 2-3 c/s activity of amplitude higher than 300 μV, from the frontal regions. Valente et al[27] described as "hypsharrhythmic-like variants" the observed EEG tracing characterized by runs of high amplitude asynchronous delta activity associated with multifocal spikes and sharp waves of moderate amplitude. This pattern, although resembling hypsharrhythmmia showed a predominance of slow waves over the epileptiform discharges. Furthermore no sleep/wake correlation was observed, while fragmentation is usually seen during sleep in hypsharrhythmia. Moreover in West syndrome periods of suppression are seen in the context of a more chaotic EEG[28].

The co-occurrence of complex absences and tonic seizures with mental retardation is also misinterpreted as Lennox-Gastaut syndrome manifestation. Once again the accurate evaluation of the EEG pattern should help differentiating these two conditions for the presence in AS of rhythmic runs of slow spike-wave complexes[25].

## Treatment

Different anticonvulsants have been used and drug resistant epilepsy described in AS patients. Today there is enough evidence that some drugs act better than others and that often, (as in RTT patients) movement abnormalities, tremors and attention deficit spells can be misinterpreted as seizures given that the EEG anomalies persist also in absence of crises.

From an accurate evaluation of 45 cases, Valente et al[30] reported a good control of crises with Valproate (VPA) alone or in association with benzodiazepines (fenobarbital PB or clonazepam CZP). Recurrent myoclonic SE were best controlled by VPA and ethosuximide. On the contrary, epilepsy was worsened by carbamazepine (CBZ), oxacarbazepine and vigabatrin[31]. Topiramate (Franz et al[32]) and ethosuximide (Sugiura et al[33]) were successful in small samples of AS patients with drug resistant epilepsy.

Dion et al[34] evaluated Lamotrigine(LMT) efficacy in 5 patients with different types of seizures (myoclonic, tonic clonic, atypical absences) unresponsive to other drugs (VPA, Benzodiazepines, phenytoine, carbamazepine and topiramate) obtaining a good control in 3 of them. The potential myoclonic effect of LMT limits its possible use in these patients, and more case controlled studies are needed.

Ketogenic diet was helpful in some children with untreatable epilepsy[4]. Piracetam is said to be effective in controlling distal myoclonus[26].

Non convulsive status epilepticus, which should be early treated according to protocols, demonstrated a variable response to treatment with benzodiazepines, ketamine and corticosteroids[35] in limited number of patients.

### Personal experience

**Figure 2:**
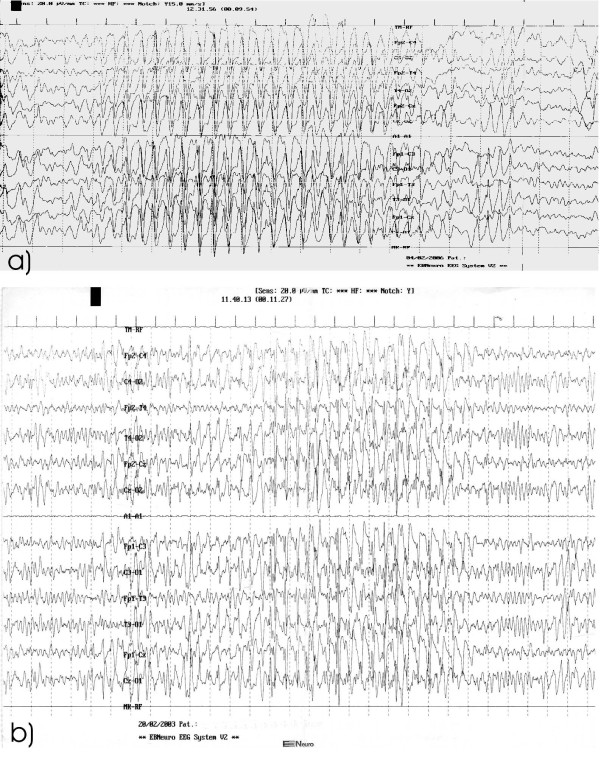
**EEG at different ages**. (a) Generalized burst of spike and spike waves with high amplitude, prevalent in the anterior regions, (b) Very slow background activity mixed with multifocal spikes.

Our series of patients with AS showed a seizures type distribution similar to that reported in Literature. All patients had febrile seizures before the onset of a frank epilepsy. Myoclonic seizures were the most frequent in infancy, followed by atypical absences and tonic clonic seizures. None had convulsive SE. Valproate and topiramate were preferentially used anticonvulsants but in the majority of cases epilepsy was hardly controlled. The critical EEG showed parossistic 1-1.5 c/s discharges of spike-waves complexes. The background activity was constituted by a persistent generalized 4-6 hz activity arising from the temporo-parietal regions (Fig. 2a). With time the EEG pattern changed showing a very slow background activity mixed with multifocal spikes (Fig. 2b).

Besides these data, our personal experience suggests us the importance of a early diagnosis in view of a possible familial recurrence. Babies with AS, are initially easily unrecognized as the facial and behaviour phenotype become clear later in life. Seizures can occur as early as the first few months and other more common diagnosis are often considered (i.e. perinatal brain hypoxia, febrile seizures, B6 unresponsive seizures). At that age, the observation of fine finger tremors may represent the only clue that should press the clinician to ask for genetic tests.

Later in life, in girls with microcephaly, seizures, stereotyped hand movements and hyperventilation Rett syndrome must be ruled out.

Nevertheless, a number of patient can remain undefined on a molecular basis. We now are aware of another condition, named Pitt-Hopkins syndrome with similar clinical signs and we suggest to verify also this possible diagnosis in AS and RTT cases without a molecular confirmation. Pitt-Hopkins syndrome, initially described in 1978 in two unrelated patients with mental retardation[36], is characterized by severe psychomotor delay, seizures and recurrent episodes of hyperventilation, acquired microcephaly and distinctive facial appearance: large nose with high philtrum, cupid's bow lips, macrostomia and wide-spaced teeth. With time a progressive protrusion of the lower face structures appear. This phenotype can easily suggest the diagnosis of AS being often associated with happy demeanor, sleep disturbances and seizures. EEG shows occipital or central delta waves unusual for the age and, in older patients, pseudoperiodic complexes during wakefulness mixed with spikes and slow spike/waves.

Pitt-Hopkins syndrome is linked to a microdeletion of chromosome 18q21 and mutations of the TCF4 gene[37]. This gene shows a widespread expression in developing human embryos playing a role on cell fate determination and differentiation during development. The impaired development of the noradrenergic neurons pathway might cause abnormal respiratory network inside the brain.

## Conclusion

Seizures are observed in the great majority of AS patients, may have a early onset and are often refractory to treatment. Atypical absences and myoclonic seizures are common as well as non convulsive status epilepticus. Different seizure types can occur in each patient. Hyperkinetic stereotypes and behavioural disturbances are at risk to be misinterpreted and considered epileptic manifestations leading to unjustified overtreatment.

Whatever is the individual natural course of seizures, EEG varies with age.

Laan et al[38] state that "*EEG abnormalities are not themselves pathognomonic of AS and have to be seen in the appropriate clinical context*". Anyhow, knowing the existence of some suggestive patterns should facilitate the early diagnosis allowing the correct genetic counselling for the family.

## Competing interests

The authors declare that they have no competing interests.

## Authors' contributions

AF and GS wrote the report. They both diagnosed index cases and followed up the patients. AP collected patients data and updated references. MC cared and performed the EEG investigations.

All Authors read and approved the final manuscript.
